# Unraveling the Signaling Networks: How Exogenous Substances Mitigate Heat Stress in Edible Fungi

**DOI:** 10.3390/jof12030220

**Published:** 2026-03-18

**Authors:** Jinjin Wen, Huilin Jing, Bin Chen, Zhenhe Wang, Jiajia Wang, Peng Yan, Chaohui Zhang, Guang Zhang

**Affiliations:** 1 School of Life Sciences, Henan Institute of Science and Technology, Xinxiang 453003, China; wen20230223@163.com (J.W.); 15939640946@163.com (H.J.); binsanity12@hist.edu.cn (B.C.); wangzhenhe1988@163.com (Z.W.); wangjiajia2420@163.com (J.W.); 2 Yuanyang County Vocational Education Center, Xinxiang 453500, China; yyy1356988@163.com

**Keywords:** heat stress, exogenous substances, edible fungi, signal transduction, mechanisms of action

## Abstract

Heat stress (HS), induced by global climate warming, is one of the major limiting factors in edible fungi production. HS suppresses mycelial growth and fruiting body formation by causing excessive accumulation of intracellular reactive oxygen species (ROS), disrupting the integrity of cell membranes and cell walls, and impairing cellular metabolism. Increasing evidence suggests that the application of exogenous substances (ESs) effectively mitigates HS in edible fungi. Based on the recent literature, this review categorizes ESs into three groups—core signaling molecules, plant growth regulators, and cytoprotective agents—and summarizes their beneficial effects against HS in edible fungi. The underlying mechanisms of ES-mediated alleviation of heat-induced damage primarily involve four pathways: (1) regulation of antioxidant systems; (2) preservation of cell wall and membrane structural integrity; (3) modulation of defense-related gene expression; and (4) regulation of carbon metabolic flux. Current challenges and corresponding strategies are discussed to provide a reference for elucidating the mechanisms by which ESs alleviate HS and to promote their practical application in edible fungi production.

## 1. Introduction

**Figure 1:**
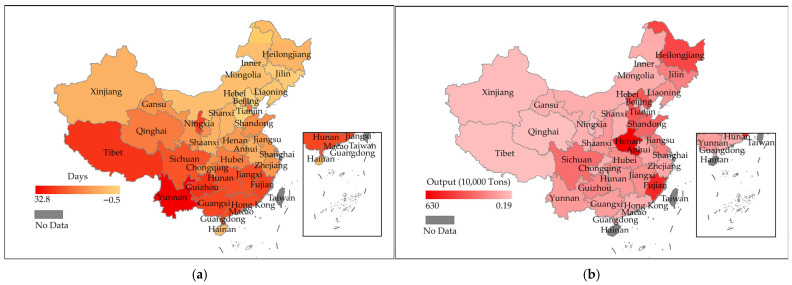
EHTDs and edible fungi production across provinces in China. (**a**) Differences in annual mean EHTDs between 2009–2023 and 1994–2008; data from Hong Kong, Macao, and Taiwan regions of China were excluded [4]. (**b**) Edible fungi production by province (region) in China in 2023; data from Hong Kong, Macao, Hainan Province, and Taiwan region of China were excluded [5].

Edible fungi constitute a group of macrofungi with significant nutritional and medicinal value, and their cultivation has become the fifth-largest agricultural sector in China [1,2]. Optimal growth of edible fungi requires suitable temperature, humidity, illumination, and gaseous conditions, among which temperature critically affects mycelial growth, fruiting body development, and yield formation [3]. However, rising global temperatures severely constrain the development of the edible fungi industry. The annual increase in extremely high-temperature days (EHTDs, defined as days when the daily maximum temperature exceeds a fixed threshold) significantly elevates the risk of HS in edible fungi. Observational data indicate that, over the past 15 years (2009–2023), the annual mean EHTDs have increased across all 30 provinces in China compared to the previous 15-year period (1994–2008) (Figure 1a). Further analysis of provincial edible fungi production data in China in 2023 (Figure 1b) has revealed that Henan, Fujian, and Heilongjiang are ranked as the top three provinces in total edible fungi yield nationwide. Corresponding increases in EHTDs indicate that these regions are particularly vulnerable to heat stress (HS) in edible fungi production.

**Figure 2:**
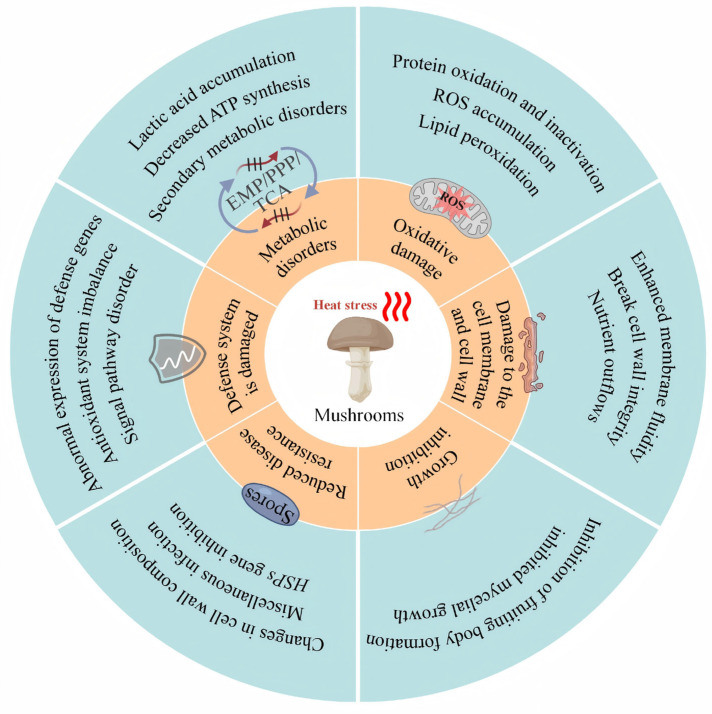
Damage caused to edible fungi by heat stress. The figure illustrates six specific effects of heat stress on edible fungi. Reduced growth and impaired disease resistance are the direct consequences of heat stress damage; other factors potentially contribute to these two primary outcomes.

HS triggers a series of harmful reactions in edible fungi, which include lipid peroxidation mediated by outbursts of reactive oxygen species (ROS) as a result of disruption of the mitochondrial electron transport chain [6], as well as protein misfolding or inhibition of the functional activity of proteins. The sum total of all these physiological disruptions leads to heat injury in edible fungi (Figure 2), which has a significant impact on the production of edible fungi [7,8]. Although edible fungi can adjust their metabolism to enhance resistance to HS [6,9,10,11,12], they still exhibit high mortality when exposed to extreme or prolonged high temperatures [13,14]. Therefore, improving thermotolerance and alleviating heat-induced injury in edible fungi has become a major focus of current research. The breeding of heat-tolerant strains is considered to be a fundamental solution, while the development of new strains is limited due to long breeding periods and genetic stability problems, which makes it complicated to acquire cultivars suitable for cultivation at high temperatures.

Exogenous substances (ESs) are compounds applied externally that influence the growth and metabolism of organisms. Increasing evidence suggests that ESs can enhance thermotolerance in edible fungi, either directly or indirectly by modulating metabolic processes [15,16,17,18,19]. Previous studies have systematically reviewed HS response mechanisms in edible fungi [20]. However, there is a notable lack of reviews on the mechanisms by which ESs mitigate HS effects. Therefore, this paper systematically summarizes four pathways by which ESs alleviate HS in edible fungi, providing valuable insights for their application in edible fungi production.

## 2. Responses of Edible Fungi to Heat Stress

HS directly affects the integrity of the mycelial cell membrane structure, cellular osmotic balance, and the accumulation of ROS, thus inhibiting mycelial growth and fruiting body formation [21]. Edible fungi have developed a series of stress responses to HS signals, which include the activation of mitogen-activated protein kinase (MAPK) signaling pathways, reinforcement of antioxidant defense, and upregulation of *Hsp* gene expression and protein synthesis. A comprehensive understanding of the mechanisms of HS responses in edible fungi will help establish a theoretical foundation for efficient heat-tolerant cultivation techniques.

### 2.1. Heat Signal Perception and Transduction

The cell membrane is a basic structural component that protects the intracellular milieu of living organisms [22]. Moreover, it plays a key role in the perception and transduction of stress signals [23,24]. Previous studies have shown that the increase in membrane fluidity induced by HS regulates secondary metabolism in *Ganoderma lucidum* and stimulates the production of Ganoderic acids (GAs) [25,26,27]. However, this research primarily emphasizes GA biosynthesis rather than strategies for improving heat tolerance in *G. lucidum*. Our understanding of how cell membranes perceive HS remains limited. The membrane contains numerous proteins, indicating that some of them may be involved in thermal signal perception. Although activation of phospholipase D (PLD) due to increased membrane fluidity has been suggested [26], the roles of other membrane-associated enzymes remain unclear and require further investigation.

**Figure 3:**
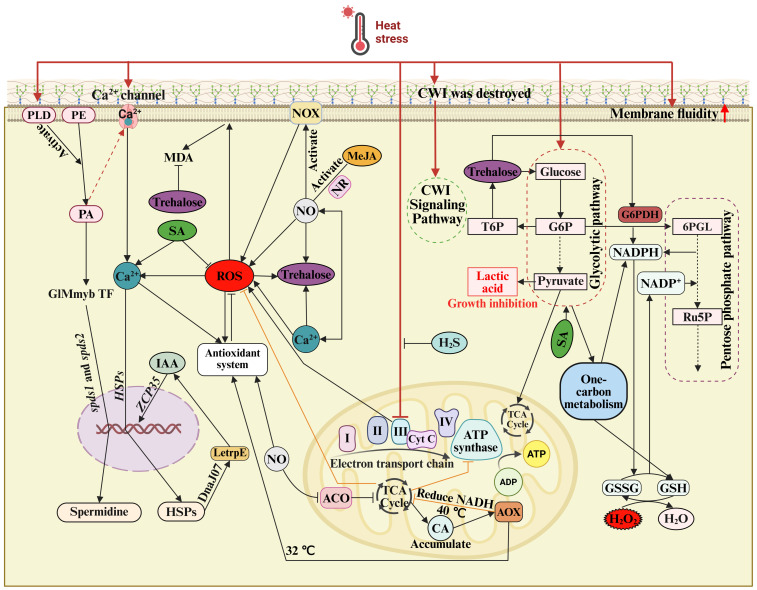
Response of edible fungi to HS. A complex HS response network operates in edible fungi. It primarily involves the antioxidant system, mitochondrial responses, regulation of defense-related gene expression, metabolic adjustments, and the cell wall integrity signaling pathway. Abbreviations: 6PGL, 6-phosphate-gluconolactone; ACO, aconitase; ADP, adenosine diphosphate; AOX, alternative oxidase; ATP, adenosine triphosphate; CA, citric acid; CWI, cell wall integrity; G6P, glucose-6-phosphate; G6PDH, glucose-6-phosphate dehydrogenase; GSH, reduced glutathione; GSSG, oxidized glutathione; H_2_O_2_, hydrogen peroxide; H_2_S, hydrogen sulfide; HSPs, heat shock proteins; IAA, indole-3-acetic acid; MDA, malondialdehyde; MeJA, Methyl jasmonate; NADP^+^, nicotinamide adenine dinucleotide phosphate; NADPH, nicotinamide adenine dinucleotide phosphate hydrogen; NO, nitric oxide; NOX, NADPH oxidase; NR, nitrate reductase; PA, phosphatidic acid; PE, phosphatidylethanolamine; PLD, phospholipase D; ROS, reactive oxygen species; Ru5P, ribose-5-phosphate; SA, salicylic acid; T6P, trehalose-6-phosphate; TCA, tricarboxylic acid; TF, transcription factors.

Heat signal transduction involves various signaling pathways, which rely on various signaling compounds, including ROS, Ca^2+^, nitric oxide (NO), carbon monoxide (CO), hydrogen sulfide (H_2_S), plant hormones, and specific transcription factors [28,29,30,31]. These components together form a complex HS signal transduction pathway in edible fungi (Figure 3).

### 2.2. MAPK Signaling Pathways in Fungi Heat Stress

The MAPK pathway mediates multiple regulatory responses to HS signals, particularly in terms of maintaining fungal cell wall stability [32,33]. Upon receiving particular signals, receptor proteins transmit the signal through a three-kinase cascade of MAPK kinase kinases (MAPKKKs), MAPK kinases (MAPKKs), and MAPKs, which allows for the signal to be amplified, transduced, and finally for the MAPKs to be activated. HS-induced cell wall damage in edible fungi rapidly activates the MAPK pathway. This pathway regulates synthesis of cell wall components and preserves cell wall integrity (CWI) [11,16]. Similar findings were reported in *P. ostreatus* and *Lepista sordida* [34,35]. iTRAQ-based quantitative proteomics further showed that MAPK expression was upregulated under HS but downregulated under normal conditions [10]. Collectively, these studies demonstrate that MAPK signaling plays a critical role in the HS response of edible fungi.

### 2.3. ROS Signaling and the Antioxidant Response

ROS play a dual role in the cellular response to HS and function as key components of the HS signaling network (Figure 3). ROS mainly include superoxide anion, peroxyl radicals, and hydrogen peroxide (H_2_O_2_) [36,37]. Current evidence suggests that damage to the mitochondrial electron transport chain induced by high temperatures represents a major source of ROS in edible fungi [6]. ROS scavenging primarily relies on enzymatic and non-enzymatic antioxidants within organisms [38], which collectively maintain intracellular ROS homeostasis. The enzymatic antioxidant system mainly includes superoxide dismutase (SOD), peroxidases (POD), and catalase (CAT), whereas non-enzymatic antioxidants comprise ascorbic acid (VC), reduced glutathione (GSH), and reduced nicotinamide adenine dinucleotide phosphate hydrogen (NADPH) [39]. Additionally, ROS accumulation increases intracellular Ca^2+^ concentrations, activating Ca^2+^ dependent signaling pathways [40]. However, ROS function is concentration-dependent. Elevated levels of ROS cause oxidative damage to cells, whereas lower levels act as signaling molecules. It remains unclear how edible fungi accurately detect ROS concentrations. They may, similarly to many organisms, rely on reversible redox modifications of cysteine residues for precise ROS sensing [41].

### 2.4. Role of Heat Shock Proteins in Alleviating Heat Stress in Edible Fungi

Heat shock proteins (HSPs) serve as downstream effectors of thermal signal transduction, accumulate rapidly, and provide an important mechanism that enables organisms to tolerate HS. HSPs are classified by molecular weight into HSP100, HSP90, HSP70, HSP60, HSP40, and small heat shock proteins [42,43]. HSPs play important roles in fungal growth, mitochondrial stability, and adenosine triphosphate (ATP) synthesis [44].

Previous studies have demonstrated that HSP20, HSP40, HSP70, and HSP90 [31,45,46,47], along with specific transcription factors [48], are involved in edible fungi HS responses. Moreover, HSPs perform distinct functions during HS responses. For instance, in *Lentinula edodes*, HSP40 (DnaJ07), alleviates HS by regulating indole-3-acetic acid (IAA) biosynthesis under high-temperature conditions [49], while overexpression of *LeHSP20* promotes growth recovery after HS [45]. Additionally, expression levels of *HSP60.5*, *HSP70.6*, *HSP90.1*, and *HSP100.1* are significantly increased in *L. edodes* under HS [50]. In model organisms, such as fission yeast, both mild HS (37 °C) and high HS (45 °C) rapidly induce the synthesis of HSP90 [51]. Collectively, these findings indicate that HSPs play a pivotal role in mitigating HS in edible fungi.

## 3. Alleviating Effects of Exogenous Substances on Heat Stress in Edible Fungi

Previous studies have systematically reviewed research progress on ESs in mitigating HS in plants [52]. However, systematic summaries regarding ESs alleviating HS in edible fungi remain relatively scarce. Therefore, this article summarizes identified ESs that alleviate HS in edible fungi and describes their role in this process (Table 1). Based on their functional characteristics, ESs involved in alleviating HS in edible fungi can be broadly classified into three categories: core signaling molecules, plant growth regulators, and cytoprotective agents. These substances mainly enhance thermotolerance in edible fungi by regulating antioxidant systems, maintaining cell wall and membrane integrity, modulating defense-related gene expression, and regulating carbon metabolic flux (Section 4).

### 3.1. Core Signaling Molecules as Exogenous Substances

NO is a small, nonpolar molecule capable of rapidly diffusing across cell membranes, thereby exerting signaling functions [69,70]. In edible fungi, two distinct hypotheses for NO biosynthesis exist: one involving nitrate reductase (NR) [18,71], and the other nitric oxide synthase (NOS) [54]. However, it remains unclear which pathway is dominant. Under non-HS conditions, Methyl jasmonate (MeJA) has been found to stimulate the production of NO through the activation of the NR pathway. NO acts as a signaling molecule upstream to stimulate NADPH oxidase (NOX), leading to increased production of ROS, which in turn leads to increased synthesis of GA and its precursors in *G. lucidum* [71]. ROS generation is not due to electron transport chain damage. Simultaneously, enhanced antioxidant enzyme activity in the mycelium effectively eliminates excess ROS, preventing oxidative damage [71]. This also indicates that NO potentially has multiple modes of action within organisms.

H_2_S has been found to be the third gaseous signaling molecule after NO and CO [72]. Research indicates that H_2_S significantly mitigates thermal damage in *G. lucidum* under HS [58]. Notably, H_2_S-mediated alleviation of HS in plants is also accompanied by the involvement of Ca^2+^ and NO signaling pathways [73,74]. However, currently no definitive studies demonstrate this phenomenon in edible fungi.

Ca^2+^ plays a vital role as a second messenger in the regulation of cells [75]. When organisms experience stress, intracellular Ca^2+^ levels increase, generating Ca^2+^ signals [76,77]. This phenomenon has been observed in *G. lucidum* under HS [19]. Furthermore, exogenous supplementation of Ca^2+^ was found to relieve the inhibitory effect of HS on mycelial growth [59]. These results suggest that Ca^2+^ plays a pivotal role in the signal transduction of HS and the alleviation of HS in edible fungi.

### 3.2. Plant Growth Regulator-Type Exogenous Substances

In edible fungi, IAA has regulatory effects in *P. sajor-caju* [78,79] and *L. edodes* [31]. However, it has not been confirmed whether IAA alleviates HS in *P. sajor-caju*. Moreover, under high-temperature conditions, expression levels of *Hsp40* (*LeDnaJ*) and the indole-3-pyruvate monooxygenase gene *LeYUCCA* are significantly higher in heat-tolerant strains of *L. edodes* than in heat-sensitive ones [60]. These studies suggest that IAA may contribute to HS alleviation in edible fungi. However, IAA is less stable than auxin analogs such as naphthaleneacetic acid (NAA) and 2,4-dichlorophenoxyacetic acid (2,4-D). 2,4-D has been demonstrated to alleviate HS in *L. edodes* [80]. Furthermore, exogenous ABA, Jasmonic acid (JA), Gibberellic acid (GA_3_), and trans-zeatin (tZ) have been reported to enhance thermotolerance in *P. ostreatus* [15]. ABA can also activate Ca^2+^ channels in *G. lucidum*, promote Ca^2+^ influx, and subsequently regulate the expression of Ca^2+^ signaling-related genes [81]. However, detailed mechanisms regarding ABA regulation of Ca^2+^ influx remain unverified.

Salicylic acid (SA) is widely considered a plant hormone involved in stress resistance, significantly enhancing plant tolerance to pathogen infections and HS [82]. Similarly, SA participates in fungal stress responses. In *P. ostreatus*, both exposure to heat at 40 °C for 24 h and exogenous treatment with 0.05 mM SA notably elevated endogenous SA levels [6,16]. Additionally, when SA was applied externally to *P. ostreatus* mycelia, it relieved damage caused by HS [16]. These studies support the fact that SA has a pivotal role to play in the regulation of HS in *P. ostreatus*, similar studies have been conducted on *P. eryngii* [61]. However, in *P. eryngii*, only limited physiological indicators were assessed, and underlying mechanisms for observed improvements were not thoroughly investigated.

### 3.3. Cell Protectant-Type Exogenous Substances

Trehalose is a well-recognized stress-related metabolite, and its role in stabilizing cellular membranes is essential [83,84,85]. Intracellular trehalose in *P. pulmonarius* increases quickly under HS at 40 °C, and supplementation of exogenous trehalose may increase the biosynthesis of trehalose [63]. Interestingly, even in the absence of trehalose synthesis genes, *Saccharomyces cerevisiae* can increase intracellular trehalose levels by absorbing exogenous trehalose [86]. Furthermore, supplementation of trehalose has been found to enhance the recovery of the mycelial growth of *P. ostreatus* under HS and reduce the inhibition of mycelial growth caused by HS [36]. However, the effects of exogenous trehalose vary among edible fungi. In *L*. *edodes*, trehalose enhances the DPPH free radical scavenging rate, thereby improving mycelial antioxidant capacity [17].

In addition to the aforementioned substances, other cellular protective agents have been reported to alleviate HS-induced damage in edible fungi (Table 1). Although considerable research has investigated ESs for alleviating HS in edible fungi, additional evidence is still required to confirm the general applicability of these substances.

## 4. Mechanisms by Which Exogenous Substances Alleviate Heat Stress in Edible Fungi

### 4.1. Regulation of Antioxidant Systems

In fungal cells, ROS are primarily produced by the mitochondrial respiratory chain. Under normal conditions, intracellular ROS levels are tightly regulated and participate in hyphal branching, growth, and differentiation [87,88,89]. However, under HS, this redox balance is disrupted, leading to excessive ROS accumulation and oxidative damage [90]. Oxidative stress is considered a major cause of HS-induced inhibition of mycelial growth in edible fungi [14]. Therefore, the development of thermotolerance largely depends on the efficient removal of ROS and the minimization of oxidative injury. The removal of ROS has been demonstrated to occur through two pathways: antioxidant enzymes and non-enzymatic antioxidants. It has been empirically proven that ESs play a crucial role in alleviating oxidative injury caused by HS through three pathways: direct scavenging of ROS, acceleration of ROS degradation, and inhibition of ROS production. NAC and VC [91] can scavenge ROS through their functional groups, thereby reducing oxidative injury. However, this pathway has certain limitations, as ESs may degrade during application and show limited uptake.

Enzymatic detoxification is considered the main pathway for the elimination of ROS [92]. Core antioxidant enzymes include SOD, CAT, ascorbate peroxidase (APX), dehydroascorbate reductase (DHAR), monodehydroascorbate reductase (MDHAR), and glutathione reductase (GR). Numerous ESs have been shown to enhance the activities of these enzymes under HS conditions (Table 1). For instance, *P. ostreatus* was subjected to HS at 40 °C and then treated with 0.01 or 0.05 mM SA. Consequently, SOD, CAT, APX, and POD enzyme activities were significantly increased, while H_2_O_2_ and malondialdehyde (MDA) contents were decreased [11]. Similar results were obtained when PABA was employed to treat *A. bisporus* [46]. Additionally, SA has been reported to alleviate heat-induced damage in *P. ostreatus* by regulating secondary metabolism [16,59].

In addition to the activation of antioxidant enzymes, ESs treatment can stimulate antioxidant synthesis. For example, *L. edodes* was treated with 5 g/L trehalose at 25 °C and showed increased polysaccharide synthesis and DPPH free radical scavenging capacity [17]. Furthermore, treatment with 0.1 mM NAC at 37 °C increased the content of reduced GSH in *L. edodes* by 31.93% [63]. Notably, oxidative damage caused by ROS is one of the major factors responsible for strain deterioration [93]. During HS, the dysfunction of the mitochondrial electron transport chain results in excessive ROS production, leading to deterioration of mitochondrial structure, which in turn enhances ROS production, thus forming a feedback loop [6,94,95]. Hence, it is clear that reduction in mitochondrial ROS production is an effective strategy to counteract HS-induced damage in edible fungi. Shangguan et al. [58] reported that NaHS treatment (H_2_S donor) improved aerobic respiration in *G. lucidum* under HS, maintained mitochondrial homeostasis, increased mitochondrial DNA copy number, and ensured efficient electron transport, thereby reducing ROS accumulation and alleviating HS-induced oxidative damage. Similar responses were also observed in *P. eryngii* var. *tuoliensi* and *G. lucidum* when treated with NO under HS [54,56]. However, the mechanisms by which NO enhances antioxidant capacity may differ among edible fungi. In *Inonotus obliquus*, NO promotes the biosynthesis of antioxidant polyphenols [96], whereas in *Flammulina velutipes*, NO enhances antioxidant enzyme activities by facilitating Ca^2+^ influx [57].

Although a large number of studies have confirmed that ESs alleviate HS in edible fungi through the regulation of ROS homeostasis, there are both common and distinct mechanisms by which different ESs regulate ROS homeostasis (Table 2). The present study mainly focuses on the effect of individual ESs on the regulation of ROS under HS conditions. Little attention has been given to the combined effects of multiple ESs. Therefore, future studies need to focus on the interaction of more than one ES in the regulation of ROS balance under HS conditions.

### 4.2. Preservation of Cell Wall and Membrane Structural Integrity

**Figure 4:**
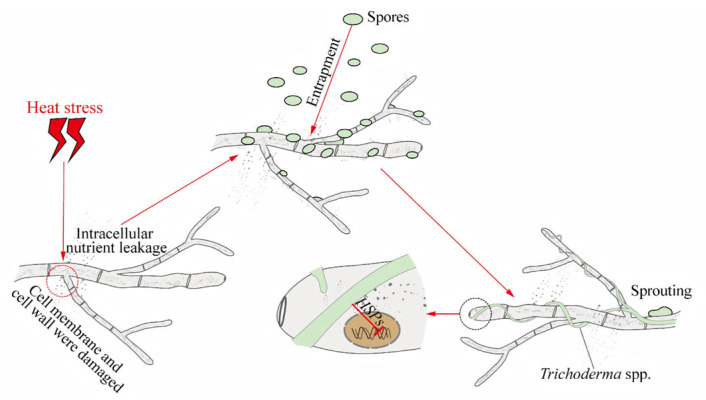
Infection of edible fungal mycelia by *Trichoderma* under heat stress. Heat stress disrupts cell permeability, leading to the leakage of nutrients from the mycelium. This creates favorable conditions for the attachment and germination of *Trichoderma* spores. Concurrently, *Trichoderma* mycelium inhibits the expression of heat shock protein genes in the edible fungi.

HS affects ROS metabolism and significantly damages cell wall and membrane integrity [97]. The fungal cell wall and membrane are key structures that perceive external temperature fluctuations and transduce stress signals [98,99,100]. The microscopic study of heat-stressed *P. ostreatus* mycelia showed that there was dehydration, shrinkage, collapse, and breaking of hyphae [6,101]. Similar results were obtained with a filamentous fungus, *Aspergillus flavus*, when it was exposed to HS [102]. Additionally, the impact of HS on cell membranes and cell walls is reflected not only in structural damage but in the weakening of the mycelial cell wall in edible fungi. This weakening increases susceptibility to degradation by enzymes such as cellulases and chitinases and reduces resistance to pathogenic fungi [103,104,105,106]. Although edible fungi normally maintain a dynamic balance with certain pathogenic or competing fungi, this balance is disrupted under HS conditions [107] (Figure 4).

Fungal cell walls have two layers: one is composed mainly of chitin and β-1,3-glucan, which are responsible for the structural integrity of the wall, while the other is involved in the interaction of the fungal cells with the environment. The two layers together form the major defense response against abiotic stresses in the fungal hyphae [108,109,110]. In the response of the cells to HS, the integrity of the cell wall is altered, as observed by the thickening of the wall, abnormal deposition of chitin, and increased porosity [104], thereby activating the CWI signaling pathway (Figure 3). In addition to chitin and β-1,3-glucan, the fungal cell walls also contain other polysaccharides, proteins, and other vital components [111]. SA modulate fungal cell wall remodeling during HS. Specifically, SA stabilizes the MAPK–Slt2 signaling pathway in *G. lucidum* [16]. As the central MAPK in the CWI pathway, Slt2 regulates cell wall biosynthesis via two major mechanisms: (i) inducing phosphorylation of transcription factors Rlm1 and SBF (Swi4/Swi6) to control chitin and β-1,3-glucan synthesis, and (ii) interacting with the target of rapamycin (TOR) pathway, which negatively regulates Slt2 signaling and influences biosynthesis of cell wall components [112,113]. Correspondingly, silencing the Slt2 gene in *G. lucidum* significantly reduced chitin and β-1,3-glucan contents [112], highlighting its crucial role in cell wall biosynthesis. Similarly, transcriptomic analysis showed that when *P. ostreatus* hyphae were exposed to HS, exogenous SA activated the MAPK pathway involved in cell wall synthesis by upregulating *Rlm1*, *Swi4*, and *Swi6*. Changes in the expression of these genes may play an important role in maintaining cell wall stability [11]. Thus, ESs may improve the resistance of edible fungi to pathogenic fungi by maintaining cell wall and membrane stability under HS. Given that edible fungi depend on coordinated activation or suppression of multiple signaling pathways to repair cell wall damage, future studies exploring ES-mediated HS alleviation should emphasize regulation at the pathway level and enhancement of recovery after stress exposure.

Heat-induced damage to membranes can be characterized as increased fluidity, structural damage, and impaired functions of proteins associated with membranes [114]. These consequences are primarily the result of the increased amounts of unsaturated fatty acids, the oxidative degradation of membrane lipids, the misfolding of proteins, and the disruption of the active site. Previous studies have indicated that ESs alleviate heat-induced membrane damage in edible fungi. For instance, the application of 2,4-D induces the increase in saturated fatty acid content in the mycelia of *L. edodes* [60], exogenous Cu^2+^ promotes membrane repair [68], and SA and NO accelerate ROS removal, reduce MDA content, and alleviate oxidative membrane damage [59,115]. Additionally, in *P. ostreatus*, Ca^2+^ triggers the synthesis of HSPs, which prevents protein misfolding and enhances membrane stability [116]. These findings suggest a potential protective mechanism against HS.

In summary, there is a considerable body of evidence that shows that various ESs play a role in the promotion of thermotolerance in fungal cell walls and membranes. However, the relationship between ESs and the signaling pathways that are involved in the repair of the cell wall and membrane under HS is not well understood. The use of multi-omics technologies is expected to improve our understanding of the role of ESs in the regulation of damage and repair mechanisms in the cell walls and membranes of edible fungi under HS.

### 4.3. Modulation of Defense-Related Gene Expression

ES alleviate HS in edible fungi not only at the physiological level but also through molecular responses. Increasing evidence suggests that edible fungi adapt to ESs by regulating the expression of their defense genes, which are related to their HS response. This includes HSP-related genes, as well as genes related to the synthesis of antioxidant enzymes and antioxidant metabolism. The transcriptional upregulation of the *Hsp* gene can be triggered by SA, ROS, Ca^2+^, and trehalose [19,117], and exogenous SA can have a particularly strong effect. Zhang et al. [59] reported that exogenous SA suppressed HS-induced *Hsp60*, *Hsp90*, and *Hsp104* expression in *P. ostreatus* by lowering intracellular ROS levels and enhancing trehalose accumulation. These findings were further validated using ROS scavengers and trehalose supplementation, confirming the ROS-degrading role of SA in fungal mycelia. Interestingly, due to functional diversity among HSPs, exogenous SA exerts dual-directional regulatory effects on *Hsp* gene expression under HS. For example, SA simultaneously downregulated two *Hsp20* genes (PLEOSDRAFT_1090983 and PLEOSDRAFT_1090314), while upregulating another *Hsp20* gene (PLEOSDRAFT_1094994) in *P. ostreatus* [11]. Notably, overexpression of *Hsp20* significantly improved thermotolerance in *L. edodes* [45]. Additionally, compounds such as 2,4-D [80], serine [118], and VC [119] promote antioxidant enzyme synthesis by activating the expression of related genes, accelerating intracellular ROS removal. Exogenous ABA-induced Ca^2+^ influx activates Ca^2+^ signaling-related genes [81], and Ca^2+^ influx also increases the expression of *Hsp60 Grp78 Hsp90*, and *Hsp104* [116]. Furthermore, GA_3_ enhances expression of *cystathionine γ-lyase* (*AbCSE*) and *cystathionine β-synthase* (*AbCBS*) genes postharvest in *A. bisporus*, thereby alleviating oxidative membrane damage via antioxidant enzyme regulation [120]. GA_3_ also enhances thermotolerance and drought resistance in *P. ostreatus* and promotes mycelial recovery after HS, although the underlying mechanisms remain unclear [15].

Transcription factors (TFs) are essential regulatory proteins that modulate gene expression. Recently, genome- and transcriptome-based studies have highlighted the crucial roles of TFs in the growth, development, and abiotic stress responses of edible fungi [121,122]. Numerous TFs, particularly heat shock transcription factors (HSFs), are involved in HS responses in edible fungi (Table 3). HSFs primarily regulate the expression of *Hsp* gene. Zhang et al. [48] reported that exogenous Cu^2+^ treatment or HS significantly enhanced *HSF2* expression in *Trametes trogii*. Notably, activation is a prerequisite for TF regulatory function. LeZCP35, a zinc finger TF belonging to the Zn_2_/Cys_6_ domain in *L. edodes*, plays an important role in heat tolerance. Silencing *LeZCP35* significantly reduces heat tolerance in *L. edodes*, and its expression is regulated by IAA (Figure 3) [123]. Apart from IAA-mediated regulation of LeZCP35, current evidence does not conclusively demonstrate direct activation of HS defense-related TFs by other ESs in edible fungi. In yeast, both Sir2 and Yap1 activate HSF1. However, Sir2-mediated activation of HSF1 induces a heat shock response, whereas Yap1-mediated activation triggers an oxidative stress response [124]. These findings suggest that the function of HSFs may depend on their specific activating factors.

### 4.4. Regulation of Carbon Metabolic Flux

The regulation of HS defense gene expression by ES directly enhances the heat resistance of fungal hyphae. Additionally, the modulation of carbon metabolic flux increases ATP content and reducing substances in hyphae under HS, thus aiding recovery from thermal damage. Central carbon metabolism (CCM) plays a fundamental role in edible fungi development, consisting primarily of the Embden–Meyerhof–Parnas (EMP) pathway, the pentose phosphate pathway (PPP), and the tricarboxylic acid (TCA) cycle. HS affects fungal CCM mainly in two ways. First, HS upregulates key EMP genes such as 6-phosphofructokinase and pyruvate kinase, accelerating glycolysis and resulting in excessive lactate accumulation. Second, HS disrupts the TCA cycle and damages the mitochondrial electron transport chain, causing excessive ROS production, reduced oxygen consumption rates, and decreased ATP synthesis. These changes collectively impair mycelial recovery following heat injury [12,137]. In *G. lucidum*, HS rapidly activates the AMPK/sucrose-nonfermenting serine-threonine protein kinase 1 (Snf1) signaling pathway, redirecting mitochondrial metabolism toward the EMP pathway. This change reduces the production of mitochondrial ROS and increases the flux in the PPP, which in turn increases NADPH synthesis [138]. Studies have shown that ESs play a key role in alleviating HS in edible fungi by regulating the distribution of carbon flux in CCM. The rate-limiting enzyme of the PPP is glucose-6-phosphate dehydrogenase (G6PDH). Under HS conditions, the level of exogenous trehalose significantly upregulates the expression of *g6pdh*, which in turn increases the synthesis of NADPH and GSH and strengthens the antioxidant capacity of mycelia [10]. Consistent with this, the overexpression of *g6pdh* increases the yield of mycelia and fruiting bodies in *Hypsizygus marmoreus* [139]. These studies highlight the important role of *g6pdh* in the growth of edible fungi, based on analyses of gene transcription and overexpression. Future studies may include silencing related genes to further clarify the regulatory role of *g6pdh* in CCM. Furthermore, exogenous plant hormones enhance yield in *P. ostreatus* [140]. SNP treatment significantly increases mycelial biomass in *F. velutipes* and *G. oregonense* [55,57]. Treatment with SA and trehalose enhances mycelial growth rates in *P. ostreatus* [29,59]. These positive outcomes likely correlate with ES-mediated CCM regulation. Hou et al. [53] found that exogenous NO reduces the expression of ACO in the TCA cycle, which reduces citric acid (CA) degradation. Accumulated intracellular CA or external CA supplementation acts as a signaling molecule that induces alternative oxidase (AOX) expression. AOX alleviates heat-induced damage in *P. ostreatus* by lowering intracellular H_2_O_2_ concentrations. However, ES can inhibit CCM activity. SA treatment under HS can inhibit CCM activity in *P. ostreatus* [11]. The metabolic pathway shifts to serine or one-carbon metabolism. This metabolic shift can stabilize oxidative phosphorylation, leading to increased ATP production and mycelial recovery from heat injury [11]. Additionally, exogenous serine supplementation enhances ATP production and antioxidant enzyme activities in *Volvariella volvacea* under non-stressed conditions [118]. This effect may arise from exogenous serine disrupting endogenous serine metabolism.

In summary, edible fungi prioritize the allocation of limited metabolic resources to defense responses during HS. However, HS-induced carbon flux redistribution often compromises growth and yield. By adjusting CCM flux partitioning, ESs promote balanced allocation of metabolic resources under HS, thereby optimizing both growth and stress defense.

## 5. Conclusions

### 5.1. Mechanistic Model of Heat Stress Alleviation by Exogenous Substances in Edible Fungi

**Figure 5:**
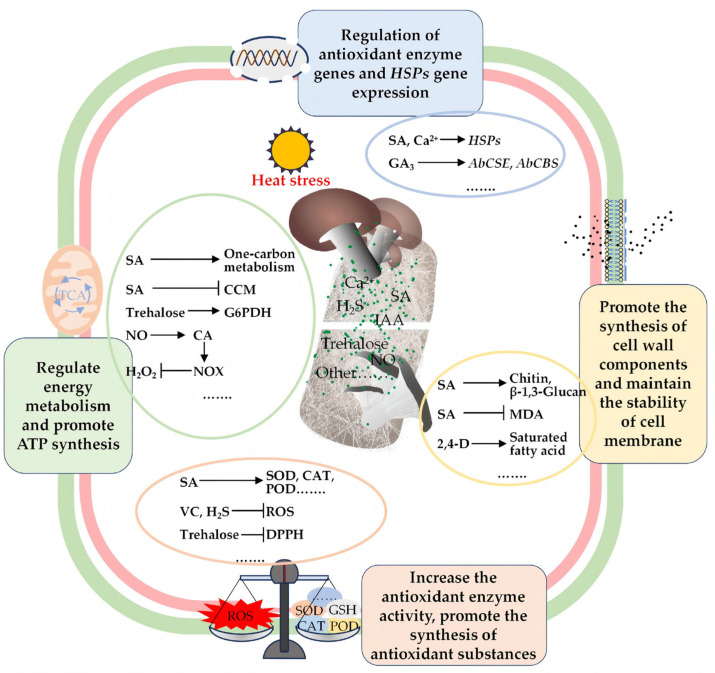
Mechanisms underlying heat stress alleviation by exogenous substances in edible fungi. The mechanisms through which exogenous substances mitigate heat stress in edible fungi are intricate. Based on fungal responses to heat stress (Figure 3), the proposed model (Figure 5) primarily involves regulation of antioxidant systems, preservation of cell wall and membrane structural integrity, modulation of defense-related gene expression, and regulation of carbon metabolic flux. Abbreviations: 2,4-D, 2,4-dichlorophenoxyacetic acid; CA, citric acid; CCM, central carbon metabolism; G6PDH, glucose-6-phosphate dehydrogenase; GA_3_, gibberellic acid; H_2_O_2_, hydrogen peroxide; H_2_S, hydrogen sulfide; HSPs, heat shock proteins; IAA, indole-3-acetic acid; MDA, malondialdehyde; NO, nitric oxide; NOX, NADPH oxidase; SA, salicylic acid; VC, ascorbic acid.

Biological processes involved in the HS response of edible fungi include antioxidant responses, expression of HS defense-related genes, and biosynthesis of defense-related metabolites and proteins. This review classifies ESs involved in edible fungi HS defense into three types: core signaling molecules, plant growth regulators, and cytoprotective agents. All three types effectively alleviate HS in edible fungi. While HS defense responses in edible fungi are mostly intrinsic, ES amplify these responses. HS initiates signaling by activating Ca^2+^ channels. This process regulates the antioxidant system and induces HSP synthesis, with ROS and Ca^2+^ acting as key signaling components, forming a complex multilevel feedback network (Figure 3). Based on this, we propose a mechanistic model (Figure 5) in which ESs alleviate HS through antioxidant responses, induction of HS defense-related genes, biosynthesis of defense-related metabolites, and regulation of carbon metabolism. This model provides improved understanding of thermotolerance mechanisms. SA, a pivotal regulator of abiotic stress responses in plants, similarly enhances HS resistance in edible fungi. In *P. ostreatus*, exogenous SA alleviates HS mainly through induction of Ca^2+^ influx, activating Ca^2+^-dependent signaling pathways, trehalose synthesis, and metabolic reprogramming, including the activation of the pentose phosphate pathway and one-carbon metabolism. These processes increase antioxidant production and reduce HS damage. Investigating SA-mediated HS alleviation mechanisms and corresponding regulatory networks will provide valuable insights into ES-mediated HS mitigation in edible fungi.

### 5.2. Major Challenges in Current Research

Most inorganic substances used to alleviate HS in edible fungi act as signaling molecules, forming complex regulatory networks (Figure 3). Due to the high diversity of edible fungi species and substantial differences in cultivation conditions, systematically identifying conserved HS response mechanisms across species remains challenging. Although current research has advanced understanding of ES roles in mitigating fungal HS, the underlying molecular mechanisms remain inadequately characterized. Comprehensive regulatory networks are incomplete, and studies targeting industrial-scale applications remain limited, restricting practical implementation. Existing studies focus on a single ES applied to a single species or strain, which limits the construction of broadly applicable regulatory networks. Second, the dosage, form of application, and timing of the ES treatments need to be optimized. At the moment, the evaluation standards for heat injury during key growth stages such as mycelial growth, primordium formation, and fruiting body development are poorly established. Moreover, the optimal growth conditions, patterns of gene expression, and HS as well as ES sensitivities vary significantly at different growth stages. Integrated assessment of the effects of safety and quality is still insufficient. On the one hand, the potential risks of ES residues in fruiting bodies and substrates are still unknown, including the potential effects on food safety and the environment. On the other hand, the potential effects of ES treatments on the nutritional value, flavor, bioactive, and medicinal compounds of fruiting bodies are still unknown and require further investigation. Regarding research methodologies, CRISPR-Cas9 technology has been explored in *A*. *bisporus* [141], *P. ostreatus* [142,143], *F. filiformis* [144], and *G. lucidum* [145]. Nevertheless, research employing CRISPR-Cas9 to enhance heat tolerance in edible fungi, and studies demonstrating ES-mediated HS mitigation using this technology remain limited.

### 5.3. Future Perspectives

Present research has created a solid theoretical basis to support the application of ESs to relieve HS in edible fungi; however, a significant gap remains between research and application. Future studies should focus on both mechanisms and application approaches to ESs relieving HS in edible fungi. Multi-omics approaches combined with gene editing tools such as CRISPR-Cas9 should be employed to explore key regulatory factors and fundamental mechanisms of ESs relieving HS in edible fungi. For example, Hou et al. [99] used transcriptomic analysis to identify significant enrichment of cell wall biosynthesis-related genes in *P. ostreatus* under HS. Lu et al. [146] combined transcriptomic and metabolomic analyses in *Auricularia heimuer* under HS, identifying 15 HS-related defense genes and three regulatory compounds. Additionally, research methods employed in model fungi, such as *S*. *cerevisiae*, may offer valuable insights, including the use of DNA microarrays [147]. In conclusion, these studies provide a basis for further elucidation of the mechanisms by which ESs alleviate HS in edible fungi.

Investigating the synergistic or antagonistic interactions of diverse ESs, together with the conserved HS signaling pathways, will help to build a comprehensive HS mitigation network. Second, the temperature sensitivity of different stages of growth needs to be characterized. Based on growth characteristics and HS-induced physiological and biochemical responses, ES types, methods, and dosages need to be optimized for each stage of growth. Third, research needs to move gradually from a controlled environment to a field or commercial production system. Currently, the majority of studies are carried out under controlled experimental conditions, while validation in a real cultivation environment is limited. Future cultivation studies must consider potential negative impacts of ESs, such as promoting harmful fungal growth and safety risks to animals. Careful consideration of precise dosages and cost-effectiveness of ESs is essential. For instance, treating *P*. *ostreatus* with cytokinins revealed that low concentrations enhance mycelial growth, whereas high concentrations inhibit growth [148]. Consequently, precise ES dosage must be carefully determined in future research. Fourth, it is important to include major targets of ESs in molecular breeding. With the help of modern biotechnologies, it becomes possible to improve endogenous heat defense systems, which will enable us to overcome the problem of HS at the very roots.

By constructing detailed regulatory networks, identifying key regulatory nodes, and considering stage-specific characteristics of fungal growth, the application of ESs can be further optimized. Greater emphasis should be placed on dynamic evaluation of ES treatments, particularly in heat-sensitive species such as *P*. *ostreatus* and *L*. *edodes*. Establishing real-time heat injury monitoring systems and determining the optimal timing for ES application under HS conditions will facilitate the development of a comprehensive ES utilization strategy. Collectively, these approaches will enhance stress tolerance in edible fungi and promote sustainable industry development.

## Figures and Tables

**Table 1:** ESs involved in alleviating heat stress in edible fungi.

Types of ESs	ESs	Target Organism	Cultivation Temperature	Application Dose	Observed Effects	References
Core signaling molecules	Nitric oxide	*P. ostreatus*	40 °C	100 μM Sodium Nitroprusside (SNP)	Reduced HS-induced ROS content by 53%, alleviating oxidative damage	[53]
		*P. eryngii* var. *tuoliensis*	37 °C	100 μM SNP	Decreased TBARS content by 51%, mitigating HS-induced membrane damage	[54]
		*G. oregonense*	32 °C	100 μM SNP	Increased mycelial biomass by 21.92%, enhancing thermotolerance	[55]
		*G. lucidum*	42 °C	500 μM SNP	Reduced mitochondrial ROS production by 60%	[56]
		*Flammulina velutipes*	37 °C	200 μM SNP	Significantly increased mycelial biomass and reduced intracellular H_2_O_2_, alleviating membrane oxidative damage	[57]
	H_2_S	*G. lucidum*	42 °C	90 μM NaHS	Increased oxygen consumption rate by 17.1% and ATP content by 29.6%	[58]
	Ca^2+^	*P. ostreatus*	40 °C	5 mM	Significantly reduced HS-induced inhibition of mycelial growth and alleviated membrane damage	[59]
Plant hormones	IAA/2,4-D	*L. edodes*	40 °C	0.01 mM	Accelerated recovery of heat-sensitive strains and enhanced thermotolerance	[60]
	Salicylic acid	*P. ostreatus*	40 °C	0.01 and 0.05 mM	H_2_O_2_ reduced to 57.6–61.2% and malondialdehyde (MDA) to 52.7–62.5% of control; SOD, CAT, and POD activities increased	[11]
		*P. eryngii*	20 °C	50 mg/L	Soluble protein and sugar contents in fruiting bodies increased by 95.5% and 41.7%, respectively	[61]
	Melatonin	*Cordyceps guangdongensis*	30 °C	10 μM	Promoted mycelial growth and enhanced HS tolerance	[62]
Cytoprotective agents	Trehalose	*P. pulmonarius*	40 °C	15/20/30 g/L	Promoted recovery from heat injury and reduced intracellular TBARS content	[63]
		*P. ostreatus*	40 °C	5/10/15 g/L	Alleviated HS-induced inhibition of mycelial growth and reduced MDA content	[36]
		*L. edodes*	25 °C	5 g/L	Increased mycelial biomass and polysaccharide content; enhanced DPPH radical scavenging activity	[17]
		*Agaricus bisporus*	28 °C	20 g/L	Enhanced mycelial thermotolerance	[64]
	Oligomycin	*P. ostreatus*	42 °C	10 μM	Reduced intracellular ROS levels and nuclear condensation	[14]
	Putrescine	*G. lucidum*	42 °C	5 mM	Increased intracellular putrescine content and regulated GAs synthesis via NO under HS	[65]
	N-acetyl cysteine	*P. ostreatus*	40 °C	4 mM	Reduced intracellular H_2_O_2_ and alleviated membrane oxidative damage	[59]
		*L. edodes*	37 °C	0.1 mM	O_2_^−^, H_2_O_2_, and TBARS reduced by 40.94%, 41.97%, and 47.62%, respectively; SOD, CAT, and POD activities significantly increased	[66]
	VC	*P. ostreatus*	40 °C	2 mM	Reduced intracellular H_2_O_2_ and alleviated membrane oxidative damage	[59]
	Gama-Aminobutyric Acid	*P. ostreatus*	35/40 °C	5–20 mM	Promoted primordium formation and development of fruiting bodies	[67]
	Para-Aminobenzoic Acid	*A. bisporus*	33 °C	10 mg/L	Reduced mycelial damage rate, increased CAT and SOD activities, and promoted HSP synthesis	[46]
	Cu^2+^	*P. ostreatus*	32 °C	200/400/600 μM	Enhanced thermotolerance, promoted mycelial growth, and protected membrane integrity	[68]

**Table 2:** Effects of exogenous substances on ROS homeostasis of edible fungi.

ESs	Source Organism	Target Pathway/Mechanism	Key Effects	References
SA	*P. ostreatus*	Antioxidant system	Increased activity of SOD, CAT, APX, GR, and POD under HS	[11,59]
		Central carbon metabolism	Increased serine synthesis and GSH production	[11]
		Mitochondrial metabolism	Reduced ROS via complex III/IV modulation	
PABA	*A. bisporus*	Antioxidant system	Increased activity of CAT and SOD under HS	[46]
NAC	*A. bisporus*	Antioxidant system	Increased activity of CAT, SOD, APX, and GPx under HS	[66]
Trehalose	*P. ostreatus*	Central carbon metabolism; antioxidant system	Elevated NADPH; promoted GSSG to GSH conversion	[12]
	*L. edodes*	Antioxidant system	Increased DPPH radical scavenging	[17]
NO	*G. lucidum*	Mitochondrial metabolism	Reduced mitochondrial ROS production under heat stress	[56]
	*F. velutipes*	Antioxidant system	Increased CAT, SOD, APX, and GPx under HS	[57]
H_2_S	*G. lucidum*	Mitochondrial metabolism	Reduced mitochondrial damage under HS	[58]

**Table 3:** Transcription factors involved in heat stress responses in edible fungi.

TFs (Family)	Source Organism	Research Technique	Primary Function	The Role/Potential Role in HS	References
Skn7	*G. Lucidum*	RNAi; qPCR	Positively regulates the expression of antioxidant enzyme-related genes and promotes cell wall component synthesis	HS significantly upregulates *GLSkn7* transcription, suggesting its involvement in HS signal transduction	[125]
MAC1	*P. ostreatus*	Phylogenetic analysis; overexpression; RNAi; qPCR	Putatively regulates copper ion transport genes and activates the antioxidant system	Overexpression of *PoMAC1a* enhances mycelial thermotolerance and recovery from heat damage; *PoMAC1b* RNAi increases thermotolerance at 32 °C; *PoMAC1a* promotes primordium formation	[126]
MYB	*P. ostreatus*	Transcriptome analysis; overexpression, RNAi; RNA-Seq, qPCR	Putatively regulates *HSPs*, *SOD*, and *CAT* genes and participates in carbon metabolism	Overexpression of *PoMYB12* and *PoMYB20* and RNAi of *PoMYB15* significantly enhance post-HS recovery; *PoMYB12* and *PoMYB20* promote growth and development, whereas *PoMYB15* inhibits growth; *PoMYB03/08/09/10* are highly expressed in spores and may be associated with spore thermotolerance	[127,128]
bZIP	*P. ostreatus*	Genome-wide identification; phylogenetic analysis; RNA-seq; RT-PCR; yeast two-hybrid assays; overexpression, RNAi	Regulates *PoHSP100* by binding to G-box (CACGTG) and C-box (CACGTC) motifs; modulates antioxidant system; affects sugar metabolism and energy supply	*PoBZIP3* overexpression markedly enhances tolerance and recovery at 40 °C; *PoBZIP3* directly interacts with *PoHSP100*; overexpression accelerates primordium and fruiting body formation; RNAi strains are more heat-sensitive; *PoBZIP3* participates in sugar metabolism, antioxidant defense, and sexual reproduction	[129]
GCN4	*G. lucidum*	RNAi, qRT-PCR, Western blot	Reduces S6K phosphorylation and suppresses amino acid anabolism	Enhances the TCA cycle and glycolysis; suggested to participate in HS-induced metabolic reprogramming	[130]
C2H2-ZFPs	*P. ostreatus*	Genome-wide identification; phylogenetic analysis; qRT-PCR	Putatively induces HSPs or protective proteins; participates in antioxidant defense, cell wall integrity maintenance, and metabolic regulation	Different C2H2-ZFP members may act coordinately to confer HS adaptation in *P. ostreatus*	[131]
bHLH	*Sanghuangporus baumii*	Genome-wide identification and expression profiling; qRT-PCR; heterologous expression in yeast	Twelve *SbbHLH* genes show differential responses to abiotic stress, with *SbbHLH3* being the most prominent	Most *SbbHLH* genes are upregulated under HS, particularly *SbbHLH3*, suggesting a role in thermotolerance regulation	[132]
Nrg1	*P. ostreatus*	Phylogenetic analysis; homologous recombination knockout; qRT-PCR	Alleviates oxidative stress and promotes cell wall component synthesis	Plays key roles in maintaining cell wall integrity and responding to oxidative and environmental stresses; likely contributes to HS tolerance	[133]
*Swi6*B	*G. lucidum*	qRT-PCR; Western blot; ChIP-qPCR	Phosphorylated *Swi6*B shows enhanced binding to the *CAT1* promoter, activating *CAT1* expression	Overexpression enhances resistance to H_2_O_2_ and activates downstream *CAT1*, suggested to protect against HS-induced oxidative stress	[134]
Ste12-like	*F. filiformis*	Overexpression; qRT-PCR; phylogenetic analysis; conserved domain prediction	Overexpression enhances abiotic stress tolerance	As a downstream transcription factor of the MAPK pathway, it is suggested to respond to HS via pheromone signaling pathways	[135]
GlMyb	*G. lucidum*	Overexpression; RNAi; Biacore; yeast one-hybrid; EMSA	Directly binds to *spds1* and *spds2* promoters and activates transcription	Enhances thermotolerance by promoting spermidine and GA biosynthesis	[27]
Mbp1	*P. ostreatus*	Phylogenetic analysis; homologous recombination knockout; qRT-PCR	Plays a key role in cell wall synthesis regulation, particularly in controlling the synthesis of β-glucan and chitin biosynthesis	Maintains CWI and oxidative stress responses; based on its role in CWI, Mbp1 is suggested to confer potential HS resistance	[136]

## Data Availability

No new data were created or analyzed in this study. Data sharing is not applicable to this article.

## Abbreviations

The following abbreviations are used in this manuscript:

2,4-D	2,4-Dichlorophenoxyacetic acid
ABA	Abscisic acid
ACO	Aconitase
AOX	Alternative oxidase
APX	Ascorbate peroxidase
ATP	Adenosine triphosphate
CA	Citric acid
CAT	Catalase
CCM	Central carbon metabolism
CO	Carbon monoxide
CWI	Cell wall integrity
DHAR	Dehydroascorbate reductase
EHTDs	Extremely high-temperature days
EMP	Embden–Meyerhof–Parnas
ESs	Exogenous substances
G6PDH	Glucose-6-phosphate dehydrogenase
GA_3_	Gibberellic acid
GAs	Ganoderic acids
GR	Glutathione reductase
GSH	Reduced glutathione
H_2_O_2_	Hydrogen peroxide
H_2_S	Hydrogen sulfide
HOG	High-osmolarity glycerol
HS	Heat stress
HSFs	Heat shock transcription factors
HSPs	Heat shock proteins
IAA	Indole-3-acetic acid
JA	Jasmonic acid
L-NAME	L-NG-nitroarginine methyl ester
MAPK	Mitogen-activated protein kinase
MDA	Malondialdehyde
MDHAR	Monodehydroascorbate reductase
MeJA	Methyl Jasmonate
NAA	Naphthaleneacetic acid
NAC	N-acetylcysteine
NADPH	Nicotinamide adenine dinucleotide phosphate Hydrogen
NO	Nitric oxide
NOS	Nitric oxide synthase
NOX	NADPH oxidase
NR	Nitrate reductase
PA	Phosphatidic acid
PABA	Para-aminobenzoic acid
PLD	Phospholipase D
POD	Peroxidases
PPP	Pentose phosphate pathway
ROS	Reactive oxygen species
SA	Salicylic acid
SNP	Sodium nitroprusside
SOD	Superoxide dismutase
TBARS	Thiobarbituric acid reactive substance
TCA	Tricarboxylic acid
TFs	Transcription factors
tZ	Trans-zeatin
VC	Ascorbic acid
